# Magneto-transport and Thermal properties of TiH diatomic molecule under the influence of magnetic and Aharonov-Bohm (AB) fields

**DOI:** 10.1038/s41598-022-19396-x

**Published:** 2022-09-14

**Authors:** C. O. Edet, R. Khordad, E. B. Ettah, S. A. Aljunid, R. Endut, N. Ali, M. Asjad, P. O. Ushie, A. N. Ikot

**Affiliations:** 1 Institute of Engineering Mathematics, Universiti Malaysia Perlis, 02600 Arau, Perlis Malaysia; 2 Faculty of Electronic Engineering Technology, Universiti Malaysia Perlis, 02600 Arau, Perlis Malaysia; 3 Department of Physics, Cross River University of Technology, Calabar, Nigeria; 4 Department of Physics, College of Sciences, Yasouj University, Yasouj, 75918 Iran; 5 Present Address: Advanced Communication Engineering (ACE) Centre of Excellence, Universiti Malaysia Perlis, 01000 Kangar, Perlis Malaysia; 6 Theoretical Physics Group, Department of Physics, University of Port Harcourt, PMB 5323 Choba, Rivers State Nigeria

**Keywords:** Atomic and molecular physics, Chemical physics, Quantum physics, Statistical physics, thermodynamics and nonlinear dynamics

## Abstract

In this study, the effects of Aharonov-Bohm (AB) and magnetic fields on the thermodynamic and magneto-transport properties of TiH diatomic molecule using the Deng-Fan potential as a model are investigated. The functional analysis approach (FAA) is used to solve the Schrodinger equation in the presence of magnetic and AB fields with Deng-Fan potential. The energy equation, as well as the wave function, have been derived. The analytic expressions for the thermo-magnetic and transport properties of the Deng-Fan potential are derived using the energy equation and the partition function. These properties obtained are thoroughly analysed utilising graphical representations. Our analysis shows that the magnetic susceptibility of the TiH exhibits a diamagnetic behaviour, and the specific heat capacity behaviour agrees with the famous Dulong-Petit law when the system is subjected to AB field variations and a fixed magnetic field. Albeit, a slight anomaly is observed in the behaviour of the specific heat capacity. Our findings will be valuable in various fields of physics, including chemical and molecular physics and condensed matter physics, where the derived models could be applied to study other diatomic molecules and quantum dots, respectively.

## Introduction

Potential functions or models are analytical representations of physical forces acting on a particle within a defined region of space. It has been used to simulate a variety of physical processes. This is because it provides a relatively cheaper technique for modelling relevant physical systems, especially when compared to experimental and advanced computational methodologies^1–5^. Modelling inter-atomic interactions in a diatomic molecular system has been done using several potential functions^1–5^. These potential functions vary depending on the nature of the interaction in the system^5–7^. Examples of such models are; harmonic oscillator, Morse potential, and several other potentials that have been proposed. Improvements to these models with multiple fitting parameters have also been recently proposed to study diverse physical systems^8–10^. These improvements have been inspired by the fact that molecular physicist has posited that potential models with numerous fitting parameter tend to agree with experimental data better than those with fewer parameters. This means that the search for new potential functions to propose will never cease^11^. One of this potential amongst the myriads of potentials that have been explored and proposed as an improvement to the potential as mentioned earlier is the Deng-Fan potential given as follows^12–14^;

1 $$V\left( r \right) = D_{e} \left( {1 - \frac{b}{{\left( {e^{\eta r} - 1} \right)}}} \right)^{2}$$

where $b = e^{{\eta r_{e} }} - 1$ $D_{e}$ is the energy of dissociation, $r_{e}$ is the equilibrium bond length, $\eta$ is the screening parameter which determines the width of the well, and $r$ is the inter-nuclear distance. It is customary to solve the relativistic and non-relativistic equations of interest with any interaction potential using advanced mathematical procedures to study these systems^3,15–19^. In modern spectroscopic studies, it has been pointed out that the Morse potential^20,21^ has a few shortfalls. Deng and Fan^22^ were motivated by these shortfalls to propose a new and improved potential called the Deng-Fan or generalized Morse potential^23^. The potential is an unsophisticated modification of the Morse potential and was presented in an attempt to find a more suitable diatomic molecular potential to describe the vibrational spectrum^24^. It is qualitatively similar to the Morse potential but has the correct asymptotic behaviour as the internuclear distance approaches zero^25^. Several non-relativistic and relativistic quantum mechanical studies have been carried out using the Deng-Fan potential^12,24,26–29^. In the light of the preceding, we are poised to study how perturbations (magnetic and Aharonov-Bohm (AB) fields) affect the thermal and magnetic properties of Titanium Hydride diatomic molecule modelled by Deng-Fan potential.

Titanium hydride (TiH) is selected in this study because of its outstanding ability of "hydrogen bonding ." Moreover, metal hydrides are generally exploited in several industrial applications, and amongst them is their hydrogen storage ability, which is one of the most significant^30–32^. It has been subjected to an intense theoretical and spectroscopic investigation, but not much information about the thermo-magnetic properties of this hydride is known. Because of the high level of interest in this molecule and its numerous uses in the industrial sector, it is necessary to investigate its transport and thermo-magnetic characteristics, particularly at higher temperatures, as well as other important features as this lacks greatly in literature.

It is established in the literature that some effects related to the magnetic field, such as; Zeeman, Rashba and Dresslhauss effects, have recently become particularly important in changing the behaviour of some quantum systems^33–36^. The magnetic field's elimination of degeneracy is notable among them^37^. Furthermore, it has recently been discovered that when the Aharonov-Bohm (AB) field is introduced to a system, it performs such a function^37–41^ Interestingly, several research focused on the impacts of this perturbing field on several systems have been presented in the past^9,16,33,42–44^. Edet et al.^45^ solved the position-dependent mass Schrödinger equation (PDMSE) for the screened Kratzer potential in the presence of AB and position-dependent external magnetic fields. Edet and Ikot^46^ studied the N_2_, I_2_, CO, NO and HCl diatomic molecules using the Hulthen-Kratzer potential (HKP) model in magnetic and AB fields. Given the preceding, this work intends to evaluate the impact of perturbing external fields on the thermodynamic and magnetic features of the Deng-Fan potential, following in the footsteps of prior investigations. As a result, the purpose of this study is to use a functional analysis method to solve the 2D SE with perturbations utilizing the Deng-Fan potential as the interaction potential. The energy derived will be used to investigate the thermal and magnetic properties of the TiH diatomic molecule, taking into account the impact of the magnetic and AB fields.

The magnetocaloric effect is another topic that is rarely investigated (MCE). MCE is the temperature variation of a material in response to a changing magnetic field. $\Delta \vec{B}$^47–49^. MCE is described by the entropy and temperature variations because it may be observed when an entropy change $\Delta S$ the material exchanges heat through a temperature change $\Delta T$^50–53^. MCE is an interesting effect and it is defined as the intrinsic property of magnetic materials which is expressed by temperature change (ΔT) of a magnetic material due to the application of a magnetic field^54,55^. Some authors have studied this concept as applied to several scenarios. Rastegar-Sedehi^33^ studied the MCE in Rashba spin–orbit coupling and Zeeman splitting of a narrow nanowire quantum dot. Rastegar-Sedehi and Khordad^56^ studied the MCE, magnetic susceptibility and specific heat of tuned quantum dot/ring systems. To the best of our knowledge no study have considered the magnetocaloric effect of TiH diatomic molecule. This gap will be addressed in this study. To this end, the goal of this paper is in three-fold; (1) Solve the SE with the Deng-Fan potential in the presence of magnetic and Aharonov-Bohm fields using the functional analysis approach (FAA). (2) obtain the partition function of the system. (3) Employ the partition function to study the MCE, thermo-magnetic and transport properties of the TiH diatomic molecule.

In view of this, this paper is organized in the following order. In “Non-relativistic model and solutions” section, the solutions of the Schrödinger equation (SE) with the Deng-Fan potential is presented. Evaluation of the partition function and thermo-magnetic properties of the system will be presented in “Magneto-transport and thermal properties of Deng-Fan potential (DFP) model” section. The application and discussion of results is presented in “Discussion and application of results” section. In “Conclusion” section, we present conclusions and future outlook.

## Non-relativistic model and solutions

Let us consider a non-relativistic system where the particle moves on a plane under the effect of Deng-Fan potential (DFP) in the presence of AB flux and an external magnetic field. In this case, one can employ stationary Schrödinger equation to investigate the system in polar coordinates

2 $$\left[ {\frac{1}{2\mu }\vec{p}^{2} - D_{e} \left( {1 - \frac{b}{{\left( {e^{\eta r} - 1} \right)}}} \right)^{2} } \right]\psi \left( {r,\varphi } \right) = E\psi \left( {r,\varphi } \right),$$

Here, $E$ denotes the energy eigenvalues, and μ indicates the effective mass of the system. In this manuscript we intend to analyze the physical process under the effect of DFP as given in Eq. (1). It is worth noting that, under such consideration the momentum operator of the charged particle has to be modified. To this end, we minimally couple a four-vector to the momentum operator as follows:

3 $$\vec{p} = \left( {i\hbar \vec{\nabla } + \frac{e}{c}\vec{A}} \right).$$

To indicate the magnetic field and AB flux together, we express the vector potential, $\vec{A}$, as a superposition of two terms as $\overrightarrow {A} = \overrightarrow {A}_{1} + \overrightarrow {A}_{2}$. Then, we assume

4 $$\overrightarrow {A}_{1} = \frac{{\vec{B}e^{ - \eta r} }}{{\left( {1 - e^{ - \eta r} } \right)}}\hat{\varphi }$$

To represent $\phi_{AB}$ flux, we take

5 $$\overrightarrow {A}_{2} = \frac{{\phi_{AB} }}{2\pi r}\hat{\varphi }$$

which satisfies $\overrightarrow {\nabla } \times \overrightarrow {A}_{1} = \overrightarrow {B}$ and $\overrightarrow {\nabla } .\overrightarrow {A}_{2} = 0$. Therefore, total vector potential reads

6 $$\vec{A} = \left( {\frac{{\vec{B}e^{ - \eta r} }}{{\left( {1 - e^{ - \eta r} } \right)}} + \frac{{\phi_{AB} }}{2\pi r}} \right)\hat{\varphi }$$

In order to solve the stationary Schrödinger equation, we make ansatz

7 $$\psi \left( {r,\varphi } \right) = \frac{1}{{\sqrt {2\pi r} }}e^{im\varphi } \tilde{F}_{nm} \left( r \right),$$

where $m$ denotes the magnetic quantum number. After we substitute Eqs. (1), (6), and (7) in Eq. (2), we get the radial equation in the form of:

8 $$\tilde{F}^{\prime\prime}_{nm} \left( r \right) + \frac{2\mu }{{\hbar^{2} }}\left[ {E_{nm} - V_{eff} \left( {r,\omega_{c} ,\xi } \right)} \right]\tilde{F}_{nm} \left( r \right) = 0$$

where $V_{eff} \left( {r,\omega_{c} ,\xi } \right)$ is the effective potential defined as follows;

9 $$\begin{aligned} V_{eff} \left( {r,\omega_{c} ,\xi } \right) & = D_{e} \left( {1 - \frac{b}{{\left( {e^{\eta r} - 1} \right)}}} \right)^{2} + \hbar \omega_{c} \left( {m + \xi } \right)\frac{{e^{ - \eta r} }}{{\left( {1 - e^{ - \eta r} } \right)r}} \\ & \,\,\,\, + \left( {\frac{{\mu \omega_{c}^{2} }}{2}} \right)\frac{{e^{ - 2\eta r} }}{{\left( {1 - e^{ - \eta r} } \right)^{2} }} + \frac{{\hbar^{2} }}{2\mu }\left[ {\frac{{\left( {m + \xi } \right)^{2} - \frac{1}{4}}}{{r^{2} }}} \right] \\ \end{aligned}$$

where $\xi = \frac{{\phi_{AB} }}{{\phi_{0} }}$ is an integer with the flux quantum $\phi_{0} = \frac{hc}{e}$ and $\omega_{c} = \frac{{e\vec{B}}}{\mu c}$ denotes the cyclotron frequency.

It is a very well known fact that, the presence of centrifugal term in the effective potential does not let us to get a solution for Eq. (9). Therefore, we employ the Greene and Aldrich approximation scheme^57^, which is only valid for small values of the screening parameter

10 $$\frac{1}{{r^{2} }} = \frac{{\eta^{2} }}{{\left( {1 - e^{ - \eta r} } \right)^{2} }}$$

to bypass the centrifugal term. Then, we introduce a new variable $s = e^{ - \eta r}$, and rewrite Eq. (9) in terms of the latter notation. We get

11 $$\frac{{d^{2} F_{nm} \left( s \right)}}{{ds^{2} }} + \frac{1}{s}\frac{{dF_{nm} \left( s \right)}}{ds} + \frac{1}{{s^{2} \left( {1 - s} \right)^{2} }}\left[ \begin{gathered} - \left( {\varepsilon - p_{0} + p_{1} + p_{3} } \right)s^{2} + \left( {2\varepsilon - p_{0} - p_{2} } \right)s \hfill \\ - \left( {\varepsilon + p_{4} } \right) \hfill \\ \end{gathered} \right]F_{nm} \left( s \right) = 0\,$$

For Mathematical simplicity, let's introduce the following dimensionless notations;

12 $$- \varepsilon = \frac{{2\mu \left( {E - D_{e} } \right)}}{{\hbar^{2} \eta^{2} }},p_{0} = \frac{{4\mu D_{e} b}}{{\hbar^{2} \eta^{2} }},p_{1} = \frac{{2\mu D_{e} b^{2} }}{{\hbar^{2} \eta^{2} }},p_{2} = \frac{{2\mu \omega_{c} }}{\hbar \eta }\left( {m + \xi } \right),\,\,\,p_{3} = \frac{{\mu^{2} \omega_{c}^{2} }}{{\hbar^{2} \eta^{2} }}\,\,{\text{and}}\,\,p_{4} = \left( {m + \xi } \right)^{2} - \frac{1}{4}$$

In order to solve Eq. (11), we have to transform the differential Eq. (11) into a form solvable by any of the existing standard mathematical techniques. Hence, we take the radial wave function of the form

13 $$\tilde{F}_{nm} \left( s \right) = s^{\lambda } \left( {1 - s} \right)^{\alpha } t_{nm} \left( s \right)$$

where

14a $$\lambda = \sqrt {\varepsilon + p_{4} }$$

14b $$\alpha = \frac{1}{2} + \sqrt {\frac{1}{4} + p_{1} + p_{2} + p_{3} + p_{4} }$$

On substitution of Eq. (13) into Eq. (11), we obtain the following hypergeometric differential equation:

15 $$s\left( {1 - s} \right)t_{nm}^{^{\prime\prime}} \left( s \right) + \left[ {\left( {2\lambda + 1} \right) - \left( {2\lambda + 2\alpha + 1} \right)s} \right]t_{nm}^{^{\prime}} \left( s \right) - \left[ {\left( {\lambda + \alpha } \right)^{2} - \left( {\sqrt {\varepsilon - p_{0} + p_{1} + p_{3} } } \right)^{2} } \right]t_{nm} \left( s \right) = 0\,$$

The form of Eq. (12) is solvable by the FAA method. However, by the quantization condition, we obtain

16 $$\left( {\lambda + \alpha } \right)^{2} - \left( {\sqrt {\varepsilon - p_{0} + p_{1} + p_{3} } } \right) = - n$$

which in turn transforms into the energy eigenvalue equation follows;

17 $$\varepsilon = - p_{4} + \frac{1}{4}\left( {\frac{{p_{0} + p_{1} + p_{3} - p_{4} - \left( {n + \frac{1}{2} + \sqrt {\frac{1}{4} + p_{1} + p_{2} + p_{3} + p_{4} } } \right)^{2} }}{{2\left( {n + \frac{1}{2} + \sqrt {\frac{1}{4} + p_{1} + p_{2} + p_{3} + p_{4} } } \right)}}} \right)$$

Substituting Eq. (12) into Eq. (17) and carrying out a simple manipulative algebra, we arrive at the energy eigenvalue equation of the EP in the presence of magnetic and AB fields in the form;

18 $$E = D_{e} + \frac{{\hbar^{2} \eta^{2} }}{2\mu }\left( {\left( {m + \xi } \right)^{2} - \frac{1}{4}} \right) - \frac{{\hbar^{2} \eta^{2} }}{8\mu }\left( {\frac{{\frac{{4\mu D_{e} b}}{{\hbar^{2} \eta^{2} }} + \frac{{2\mu D_{e} b^{2} }}{{\hbar^{2} \eta^{2} }} + \frac{{\mu^{2} \omega_{c}^{2} }}{{\hbar^{2} \eta^{2} }} - \left( {\left( {m + \xi } \right)^{2} - \frac{1}{4}} \right) - \left( {n + \tilde{\rm K}} \right)^{2} }}{{\left( {n + \tilde{\rm K}} \right)}}} \right)^{2}$$

where

19 $$\tilde{\rm K} = \frac{1}{2} + \sqrt {\frac{{2\mu D_{e} b^{2} }}{{\hbar^{2} \eta^{2} }} + \frac{{2\mu \omega_{c} }}{\hbar \eta }\left( {m + \xi } \right) + \frac{{\mu^{2} \omega_{c}^{2} }}{{\hbar^{2} \eta^{2} }} + \left( {m + \xi } \right)^{2} } .$$

20 $$\tilde{F}_{nm} \left( s \right) = N_{nm} s^{\lambda } \left( {1 - s} \right)^{\alpha } {}_{2}F_{1} \left( { - n,2\left( {\lambda + \alpha } \right) + n;2\lambda + 1;s} \right)$$

where $N_{nm}$ is the normalization constant and ${}_{2}F_{1} \left( { - n,2\left( {\lambda + \eta } \right) + n;2\lambda + 1;s} \right)$ is the hypergeometric function.

## Magneto-transport and Thermal properties of Deng-Fan potential (DFP) model

On obtaining the approximate-analytic energy Eq. (18), we can proceed to obtain the partition function and other thermodynamic functions. The partition function $Z\left( \beta \right)$ of the DFP at finite temperature $T$ is obtained using the Boltzmann factor as^4,58,59^;

21 $$Z\left( \beta \right) = \sum\limits_{n = 0}^{\omega } {e^{{ - \beta E_{n} }} }$$

with $\beta = \frac{1}{kT}$ and with $k$ is the Boltzmann constant.

Substituting Eq. (18) in (21), we have;

22 $$Z\left( \beta \right) = \sum\limits_{n = 0}^{\omega } {e^{{ - \beta \left( {\Omega_{0} - \Omega_{1} \left( {\frac{{\Omega_{2} - \left( {n + {\rm K}} \right)^{2} }}{{\left( {n + \tilde{\rm K}} \right)}}} \right)^{2} } \right)}} }$$

where $n$ is the vibrational quantum number, $n = 0,1,2,3...\omega$ , $\omega$ denotes the upper bound vibration quantum number. We have introduce the following notations:

23 $$\begin{aligned} \Omega_{0} & = \frac{{\hbar^{2} \alpha^{2} }}{2\mu }\left( {\left( {m + \xi } \right)^{2} - \frac{1}{4}} \right);\Omega_{1} = \frac{{\hbar^{2} \eta^{2} }}{8\mu }; \\ \Omega_{2} & = \frac{{4\mu D_{e} b}}{{\hbar^{2} \eta^{2} }} + \frac{{2\mu D_{e} b^{2} }}{{\hbar^{2} \eta^{2} }} + \frac{{\mu^{2} \omega_{c}^{2} }}{{\hbar^{2} \eta^{2} }} - \left( {\left( {m + \xi } \right)^{2} - \frac{1}{4}} \right); \\ \end{aligned}$$

The maximum value $$n_{\max }$$ can be obtained by setting $${\raise0.7ex\hbox{${dE_{n} }$} \!\mathord{\left/ {\vphantom {{dE_{n} } {dn}}}\right.\kern-\nulldelimiterspace} \!\lower0.7ex\hbox{${dn}$}} = 0$$ ,

24 $$n_{\max } = - \tilde{\rm K} \pm \sqrt {\Omega_{2} }$$

Replacing the summation in (22) by an integral, we have;

25 $$Z\left( \beta \right) = \int\limits_{0}^{\omega } {e^{{ - \beta \left( {\Omega_{0} - \Omega_{1} \left( {\frac{{\Omega_{2} - \left( {n + \tilde{\rm K}} \right)^{2} }}{{\left( {n + \tilde{\rm K}} \right)}}} \right)^{2} } \right)}} } dn$$

If we set $\Lambda = n + \tilde{\rm K}$, we can rewrite the above integral in Eq. (25) as follows;

26 $$Z\left( \beta \right) = \int\limits_{{q_{1} }}^{{q_{2} }} {e^{{\beta \left( {\frac{{\Omega_{1} \Omega_{2}^{2} }}{{\Lambda^{2} }} + \Omega_{1} \Lambda^{2} - \Omega_{3} } \right)}} d\Lambda }$$

27 $${\text{where}}\,\,q_{1} = \tilde{\rm K},\,\,q_{2} = \omega +\tilde{\rm K}\,\,{\text{and}}\,\,\Omega_{3} = 2\Omega_{2} \Omega_{1} + \Omega_{0}.$$

On evaluating the integral in Eq. (26), we obtain the partition function of the DFP in magnetic and AB fields as follows;

28 $$Z\left( \beta \right) = - \frac{{e^{{ - 2\sqrt { - \beta \,\Omega_{2} } \sqrt { - \beta \,\Omega_{1} \Omega_{2}^{2} } + \beta \,\Omega_{3} }} \sqrt \pi \left( \begin{gathered} Erf\left[ {q_{1} \sqrt { - \beta \,\Omega_{2} } - \frac{{\sqrt { - \beta \,\Omega_{1} \,\Omega_{2}^{2} } }}{{q_{1} }}} \right] + e^{{4\sqrt { - \beta \,\Omega_{2} } \sqrt { - \beta \,\Omega_{1} \,\Omega_{2}^{2} } }} Erf\left[ {q_{1} \sqrt { - \beta \,\Omega_{2} } + \frac{{\sqrt { - \beta \,\Omega_{1} \,\Omega_{2}^{2} } }}{{q_{1} }}} \right] - Erf\left[ {q_{2} \sqrt { - \beta \,\Omega_{2} } - \frac{{\sqrt { - \beta \,\Omega_{1} \,\Omega_{2}^{2} } }}{{q_{2} }}} \right] \hfill \\ - e^{{4\sqrt { - \beta \,\Omega_{2} } \sqrt { - \beta \,\Omega_{1} \Omega_{2}^{2} } }} Erf\left[ {q_{2} \sqrt { - \beta \,\Omega_{2} } + \frac{{\sqrt { - \beta \,\Omega_{1} \Omega_{2}^{2} } }}{{q_{2} }}} \right] \hfill \\ \end{gathered} \right)}}{{4\sqrt { - \beta \,\Omega_{2} } }}$$

### Free energy

The Helmholtz free energy is a thermodynamic potential that determines an estimate of the useful work obtained from a thermodynamic system that is closed and maintained at a constant temperature^59^. The free energy is computed using the expression given below^17^;

29 $$F\left( \beta \right) = - \frac{1}{\beta }\ln Z\left( \beta \right),$$

Substituting Eq. (28) into Eq. (29), we obtain the free energy for the TiH diatomic molecule modelled by the Deng-Fan potential as follows;

30 $$F\left( \beta \right) = - \frac{{\ln \left( { - \frac{{{\text{e}}^{{ - 2\sqrt { - \beta \,\Omega_{2} } \sqrt { - \beta \,\Omega_{1} \Omega_{2}^{2} } + \beta \,\Omega_{3} }} \sqrt \pi \left( \begin{gathered} Erf\left[ {q_{1} \sqrt { - \beta \,\Omega_{2} } - \frac{{\sqrt { - \beta \,\Omega_{1} \,\Omega_{2}^{2} } }}{{q_{1} }}} \right] + {\text{e}}^{{4\sqrt { - \beta \,\Omega_{2} } \sqrt { - \beta \,\Omega_{1} \,\Omega_{2}^{2} } }} Erf\left[ {q_{1} \sqrt { - \beta \,\Omega_{2} } + \frac{{\sqrt { - \beta \,\Omega_{1} \,\Omega_{2}^{2} } }}{{q_{1} }}} \right] - Erf\left[ {q_{2} \sqrt { - \beta \,\Omega_{2} } - \frac{{\sqrt { - \beta \,\Omega_{1} \,\Omega_{2}^{2} } }}{{q_{2} }}} \right] \hfill \\ - {\text{e}}^{{4\sqrt { - \beta \,\Omega_{2} } \sqrt { - \beta \,\Omega_{1} \Omega_{2}^{2} } }} Erf\left[ {q_{2} \sqrt { - \beta \,\Omega_{2} } + \frac{{\sqrt { - \beta \,\Omega_{1} \Omega_{2}^{2} } }}{{q_{2} }}} \right] \hfill \\ \end{gathered} \right)}}{{4\sqrt { - \beta \,\Omega_{2} } }}} \right)}}{\beta }.$$

### Entropy

Entropy is the measure of the amount of a system's thermal energy per unit temperature that cannot be used to perform any productive work. The amount of entropy in a system can be thought of as a measure of the molecular disorder, or unpredictability, of the system as a whole due to the fact that work is obtained from the orderly motion of molecules. The idea of entropy offers profound insight into the course of spontaneous change for a wide variety of phenomena encountered daily^58,60^. The entropy is computed using the expression given as^60^;

31 $$S\left( \beta \right) = \ln Z\left( \beta \right) - \beta \frac{d\ln Z\left( \beta \right)}{{d\beta }},$$

Substituting Eq. (28) into Eq. (31), we obtain the entropy for the TiH diatomic molecule modelled by the Deng-Fan potential as follows;

32 $$S\left( \beta \right) = \frac{{\left( { - 4{\text{e}}^{{2\sqrt { - \beta \,\Omega_{2} } \sqrt { - \beta \,\Omega_{1} \,\Omega_{2}^{2} } }} \left( {{\text{e}}^{{\frac{{\beta \,\Omega_{2} \left( {q_{1}^{4} + \Omega_{1} \Omega_{2} } \right)}}{{q1^{2} }}}} q_{1} - {\text{e}}^{{\frac{{\beta \,\Omega_{2} \left( {q_{2}^{4} + \Omega_{1} \Omega_{2} } \right)}}{{q_{2}^{2} }}}} q_{2} } \right)\sqrt { - \beta \,\Omega_{2} } + \sqrt \pi \left( {{\rm N}_{1} } \right){\rm T}_{1} - {\text{e}}^{{4\sqrt { - \beta \,\Omega_{2} } \sqrt { - \beta \,\Omega_{1} \Omega_{2}^{2} } }} \sqrt \pi \left( {N_{2} } \right)T_{1} + \sqrt \pi \left( {\left( {N_{3} } \right)T_{2} + {\text{e}}^{{4\sqrt { - \beta \,\Omega_{2} } \sqrt { - \beta \,\Omega_{1} \Omega_{2}^{2} } }} \left( {N_{2} } \right)T_{2} } \right)} \right)}}{{\left( {2\sqrt \pi \left( { - 1 + T_{1} + {\text{e}}^{{4\sqrt { - \beta \,\Omega_{2} } \sqrt { - \beta \,\Omega_{1} \Omega_{2}^{2} } }} \left( {T_{1} - T_{2} } \right) + T_{3} } \right)} \right)}} + \Xi$$

where

32a $$T_{1} = Erf\left[ {q_{1} \sqrt { - \beta \,\Omega_{2} } - \frac{{\sqrt { - \beta \,\Omega_{1} \Omega_{2}^{2} } }}{{q_{1} }}} \right]$$

32b $$T_{2} = Erf\left[ {q_{2} \sqrt { - \beta \,\Omega_{2} } - \frac{{\sqrt { - \beta \,\Omega_{1} \Omega_{2}^{2} } }}{{q_{2} }}} \right]$$

32c $$T_{3} = Erfc\left[ {q_{2} \sqrt { - \beta \,\Omega_{2} } - \frac{{\sqrt { - \beta \,\Omega_{1} \Omega_{2}^{2} } }}{{q_{2} }}} \right]$$

32d $$T_{4} = Erfc\left[ {q_{2} \sqrt { - \beta \,\Omega_{2} } - \frac{{\sqrt { - \beta \,\Omega_{1} \Omega_{2}^{2} } }}{{q_{2} }}} \right]$$

32e $$N_{1} = 1 + 4\sqrt { - \beta \,\Omega_{2} } \sqrt { - \beta \,\Omega_{1} \Omega_{2}^{2} } - 2\beta \,\Omega_{3}$$

32f $$N_{2} = - 1 + 4\sqrt { - \beta \,\Omega_{2} } \sqrt { - \beta \,\Omega_{1} \Omega_{2}^{2} } + 2\beta \,\Omega_{3}$$

32g $$N_{3} = - 1 - 4\sqrt { - \beta \,\Omega_{2} } \sqrt { - \beta \,\Omega_{1} \Omega_{2}^{2} } + 2\beta \,\Omega_{3}$$

32h $$\Xi = \ln \left[ {\frac{{{\text{e}}^{{ - 2\sqrt { - \beta \,\Omega_{2} } \sqrt { - \beta \,\Omega_{1} \Omega_{2}^{2} } + \beta \,\Omega_{3} }} \sqrt \pi \left( { - T_{1} + T_{2} + {\text{e}}^{{4\sqrt { - \beta \Omega_{2} } \sqrt { - \beta \,\Omega_{1} \Omega_{2}^{2} } }} \left( { - T_{1} + T_{2} } \right)} \right)}}{{4\sqrt { - \beta \,\Omega_{2} } }}} \right] $$

### Internal energy

The energy contained within a thermodynamic system is referred to as its internal energy. Internal energy is constant in an isolated system. It is the energy required to develop or prepare the system in its current internal state. It does not contain the system's overall kinetic energy, but it includes the kinetic energy of the particles inside the system. It keeps track of the system's energy gains and losses due to changes in its internal condition^45,59^. The internal energy can be evaluated using the expression below^36^;

33 $$U\left( \beta \right) = - \frac{d\ln Z\left( \beta \right)}{{d\beta }},$$

Substituting Eq. (28) into Eq. 33), we obtain the internal energy for the TiH diatomic molecule modelled by the Deng-Fan potential as follows;

34 $$U\left( \beta \right) = \frac{{\left( { - 4{\text{e}}^{{2\sqrt { - \beta \Omega_{2} } \sqrt { - \beta \Omega_{1} \Omega_{2}^{2} } }} \left( {{\text{e}}^{{\frac{{\beta \Omega_{2} \left( {q_{1}^{4} + \Omega_{1} \Omega_{2} } \right)}}{{q_{1}^{2} }}}} q_{1} - {\text{e}}^{{\frac{{\beta \,\Omega_{2} \left( {q_{2}^{4} + \Omega_{1} \Omega_{2} } \right)}}{{q_{2}^{2} }}}} q_{2} } \right)\sqrt { - \beta \,\Omega_{2} } + \sqrt \pi \left( {N_{1} } \right)T_{1} - {\text{e}}^{{4\sqrt { - \beta \,\Omega_{2} } \sqrt { - \beta \,\,\Omega_{1} \Omega_{2}^{2} } }} \sqrt \pi \left( { - 1 + 4\sqrt { - \beta \,\Omega_{2} } \sqrt { - \beta \,\Omega_{1} \Omega_{2}^{2} } + 2\beta \,\Omega_{3} } \right)T_{1} + \sqrt \pi \left( {\left( {N_{3} } \right)T_{2} + {\text{e}}^{{4\sqrt { - \beta \,\Omega_{2} } \sqrt { - \beta \,\Omega_{1} \Omega_{2}^{2} } }} \left( {N_{2} } \right)T_{2} } \right)} \right)}}{{\left( {2\sqrt \pi \beta \left( { - 1 + T_{1} + {\text{e}}^{{4\sqrt { - \beta \,\Omega_{2} } \sqrt { - \beta \,\Omega_{1} \Omega_{2}^{2} } }} \left( {T_{1} - T_{2} } \right) + T_{4} } \right)} \right)}}$$

### Specific heat capacity

The specific heat capacity of material in thermodynamics is the heat capacity of a sample of the substance divided by its mass, also known as massic heat capacity. Loosely, the quantity of heat must be added to one unit of mass of the substance to generate one unit of temperature increase. Specific heat capacity frequently changes with temperature and depends on the state of materials^56,61^. Substituting Eq. (28) into Eq. (33), we obtain the specific heat capacity for the TiH diatomic molecule modelled by the Deng-Fan potential as follows^56,61^;

35 $$C\left( \beta \right) = \beta^{2} \frac{{d^{2} \ln Z\left( \beta \right)}}{{d\beta^{2} }},$$

However, due to the complicated nature of the analytical expressions, the graphic representations are shown.

### Magnetic properties at $T \ne 0$

In this section, the magnetic properties of TiH diatomic molecule at finite temperature are considered;

#### Magnetization

Magnetization, also known as magnetic polarisation, is a vector quantity that represents the density of permanent or induced dipole moments in a magnetic material. As we know, magnetization is caused by the magnetic moment, which is caused by the mobility of electrons in atoms or the spin of electrons or nuclei. It is a very important quantity. Substituting Eq. (28) into Eq. (33), the magnetization at finite temperature for the TiH diatomic molecule modelled by the Deng-Fan potential is evaluated using the expression given below^56,56,61^;

36 $$M\left( \beta \right) = \frac{1}{\beta }\left( {\frac{1}{Z\left( \beta \right)}} \right)\left( {\frac{\partial }{{\partial \vec{B}}}Z\left( \beta \right)} \right).$$

#### Magnetic susceptibility

Magnetic susceptibility is a measure of how much a substance will become magnetized in an applied magnetic field in electromagnetism. This quantity allows a straightforward categorization of most materials' responses to an applied magnetic field into two categories: alignment with the magnetic field called, paramagnetism, or alignment against the field called diamagnetism. Substituting Eq. (28) into Eq. (37), the magnetic susceptibility at finite temperature for the TiH diatomic molecule modelled by the Deng-Fan potential is evaluated using the expression given below^56,56,61^;

37 $$\chi_{m} \left( \beta \right) = \frac{\partial M\left( \beta \right)}{{\partial \vec{B}}}.$$

#### Persistent current

A persistent current is a thermodynamic quantity. It is the continuous electric current that does not require an external power source. Such a current is usually said to be unachievable in standard electrical equipment since all commonly used conductors have a non-zero resistance, and any such current would quickly dissipate as heat. However, due to quantum phenomena, persistent currents are feasible and observed in superconductors and some mesoscopic devices. Because of size effects in resistive materials, persistent currents can emerge in microscopic samples. Persistent currents are commonly employed in superconducting magnets. Substituting Eq. (28) into Eq. (38), the persistent current at finite temperature for the TiH diatomic molecule modelled by the Deng-Fan potential is evaluated using the expression given below^56,56,61^;

38 $$I\left( \beta \right) = - \frac{e}{hc}\frac{\partial F\left( \beta \right)}{{\partial m}}.$$

Due to the complicated nature of the analytical expressions, the graphic representations are shown for the magnetic properties at finite temperature.

### Magnetic properties at $T = 0$

In this section, the expressions for evaluating the magnetic properties at zero temperature are presented below.

#### Magnetization

The magnetization at zero temperature is evaluated at zero temperature using the expresssion given below and Eq. (18)^45^.

39 $$M_{nm} = - \frac{\partial E}{{\partial \vec{B}}}.$$

#### Magnetic susceptibility

The magnetic susceptibility at zero temperature is evaluated at zero temperature using the expression given below and Eq. (18)^45^.

40 $$\chi_{m} = \frac{{\partial M_{nm} }}{{\partial \vec{B}}}.$$

#### Persistent current

The persistent current at zero temperature is evaluated at zero temperature using the expresssion given below and Eq. (18)^62^.

41 $$I_{nm} = - \frac{\partial E}{{\partial \phi_{AB} }}$$

Again, we point out here that due to the complicated nature of the expression, the graphical representations are presented.

### Magnetic entropy change

The magneto temperature effect (MCE) is the sensitivity of a magnetic material to an applied magnetic field at a specific temperature. The ability of magnetic materials to regulate their temperature or swap heat with a thermal reservoir in response to a changing magnetic field is a remarkable attribute of magnetic materials. The magnetocaloric effect is studied by considering the magnetocaloric potential (S) and the change in temperature (T). Because of this, we must first determine the entropy both with and without a magnetic field to compute the magnetic entropy change. To calculate S in an isothermal process, one uses the formula. To calculate the magnetic entropy change, the entropy is first calculated with and without magnetic field. For an isothermal process, the quantity ΔS is given by^40,56,63^:

42 $$\Delta S = S\left( {B \ne 0,T} \right) - S\left( {B = 0,T} \right).$$

## Discussion and application of results

In this section, we have carried out the numerical calculations for a TiH diatomic molecule. We use the following fitting parameters;$D_{e} = {2}{\text{.05}}$, *r*_*e*_= 1.781 Å, *η*= 1.32408 Å^−1^ and $\mu = {0}{\text{.987371}}\,amu$^64^.

**Figure 1:**
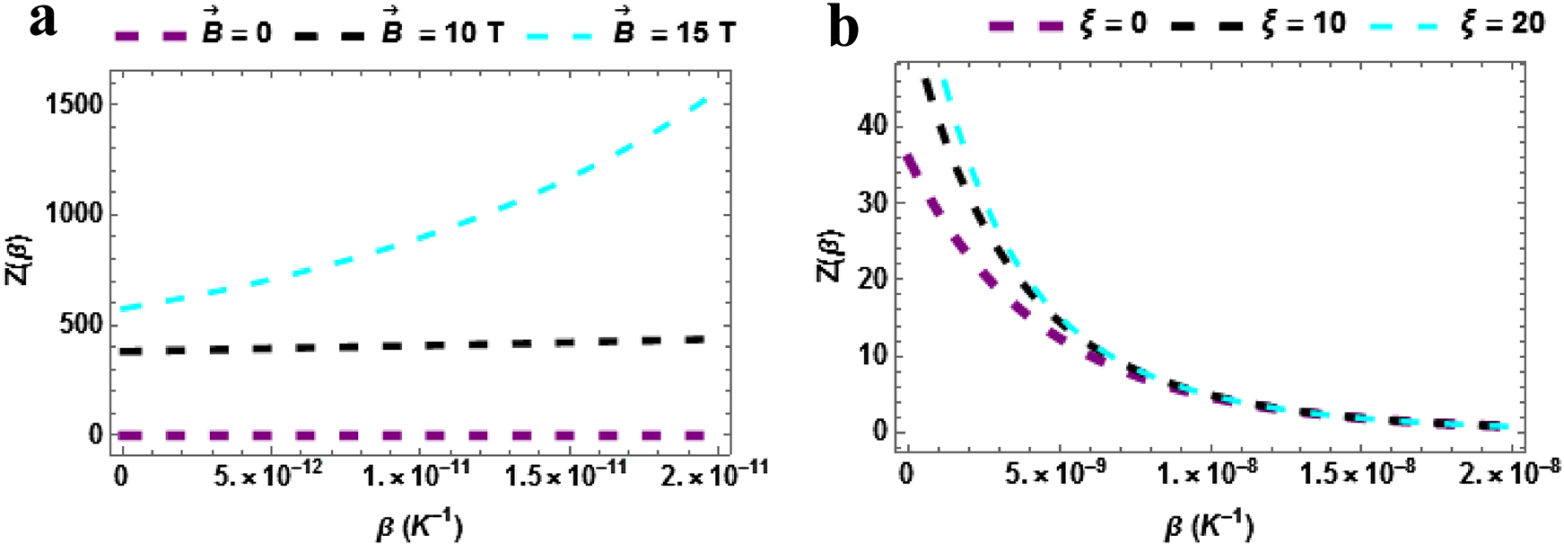
(**a**) Partition as a function of $\beta$ varying magnetic field (**b**) Partition as a function of $\beta$ varying AB field.

In Fig. 1a, the partition function is plotted against $\beta \left( {K^{ - 1} } \right)$ with varying magnetic field $\left( {\vec{B}\left( T \right)} \right)$. The partition function increases with temperature when $\vec{B} = 15T$ but shows a quasi-constant (invariant) trend when $\vec{B} = 0\,\& \,\vec{B} = 10\,T$. This means that to raise the partition function, the magnetic field must be sufficiently high. In Fig. 1b, the partition function is plotted against $\beta \left( {K^{ - 1} } \right)$ with varying Aharonov-Bohm (AB) field $\left( \xi \right)$. The partition function decreases with temperature when $\xi = 0,\,\,\xi = 10\,T\,\& \xi = 20$. The three cases considered shows a uniform influence of the AB field on the partition function.

**Figure 2:**
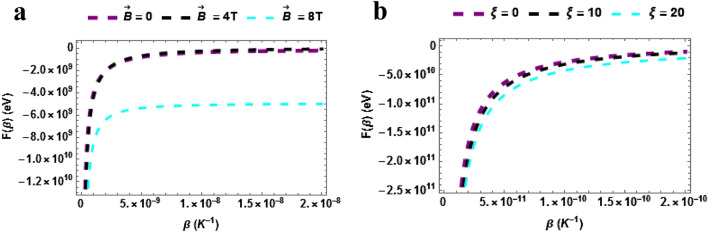
(**a**) Free energy as a function of $\beta$ varying magnetic field (**b**) Free energy as a function of $\beta$ varying AB field.

Figure 2) shows the plot of the free energy versus temperature with varying magnetic field. The free energy upsurges in a monotonic manner with rising temperature. It is clearly seen that the free energy was higher when the magnetic field is low and the converse is true. This means that the free energy of the $TiH$ rises when the magnetic field is small. Figure 2b shows the plot of the free energy versus temperature with varying AB field. The free energy upsurges in a monotonic manner with rising temperature. Again a uniform influence of the AB field on the free energy is seen for $\xi = 0,\,\,\xi = 10\,T\,\& \xi = 20$.

**Figure 3:**
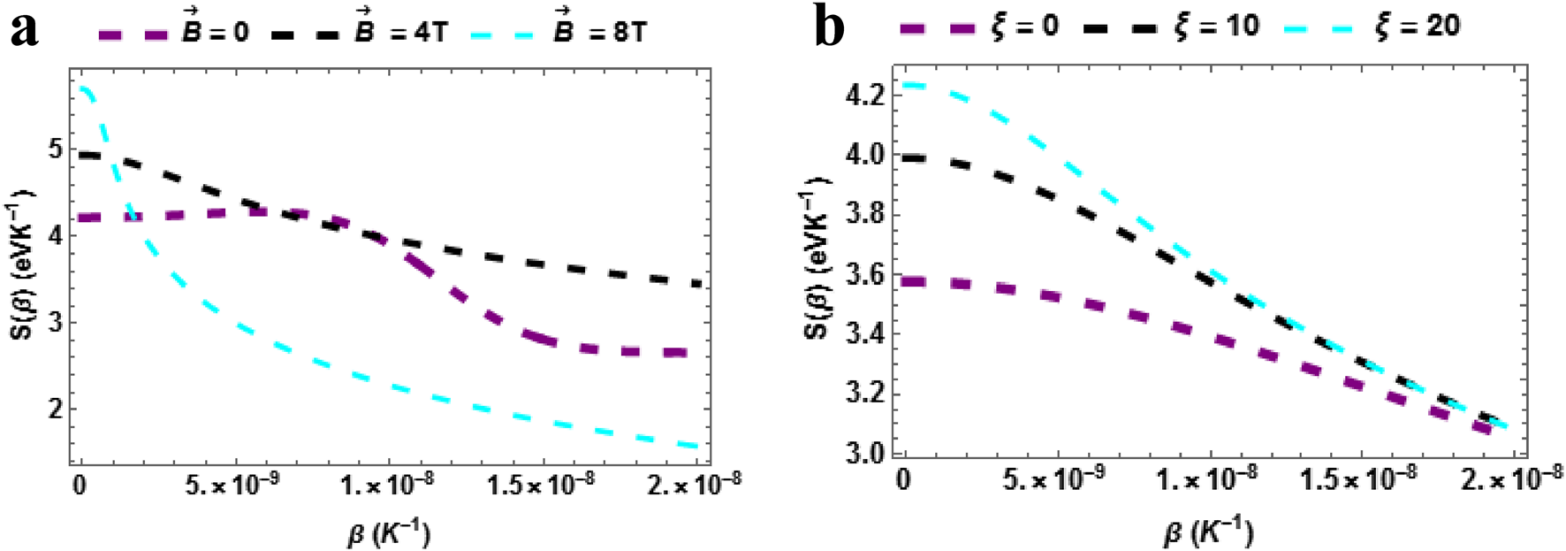
(**a**) Entropy as a function of $\beta$ varying magnetic field (**b**) Entropy as a function of $\beta$ varying AB field.

Figure 3a shows the plot a of entropy of the $TiH$ versus temperature with varying magnetic field. The entropy of $TiH$ decreases with rising $\beta \left( {K^{ - 1} } \right)$ when $\vec{B} = 8\,T$. When $\vec{B} = 0\,\& \,\vec{B} = 4T$, the entropy also decreases with surging temperature but shifts slightly above $\vec{B} = 8\,T$. However, on average, it won't be out of place to posit that a low magnetic field is required to increase the entropy of the $TiH$ diatomic molecule. To further buttress our point on the physical picture of this result, we note here that on application a magnetic field, the magnetic moments of TiH is able to lower the energy thereby leading to a lower entropy state where it is magnetized. In this case, the entropy decreases as magnetic field increase, at least when it is able to give away entropy to its environment. This effect is very important in magnetic refrigeration because the opposite is also true: a paramagnetic material tends to absorb entropy as the field is decreased.

Figure 3b shows the plot a of entropy of the $TiH$ versus temperature with varying AB field. The entropy of $TiH$ decreases with rising $\beta \left( {K^{ - 1} } \right)$ when $\xi = 0,\,\,\xi = 10\,T\,\& \xi = 20$. However, on average, it won't be out of place to posit that the higher the AB field higher the entropy of the $TiH$ diatomic molecule.

**Figure 4:**
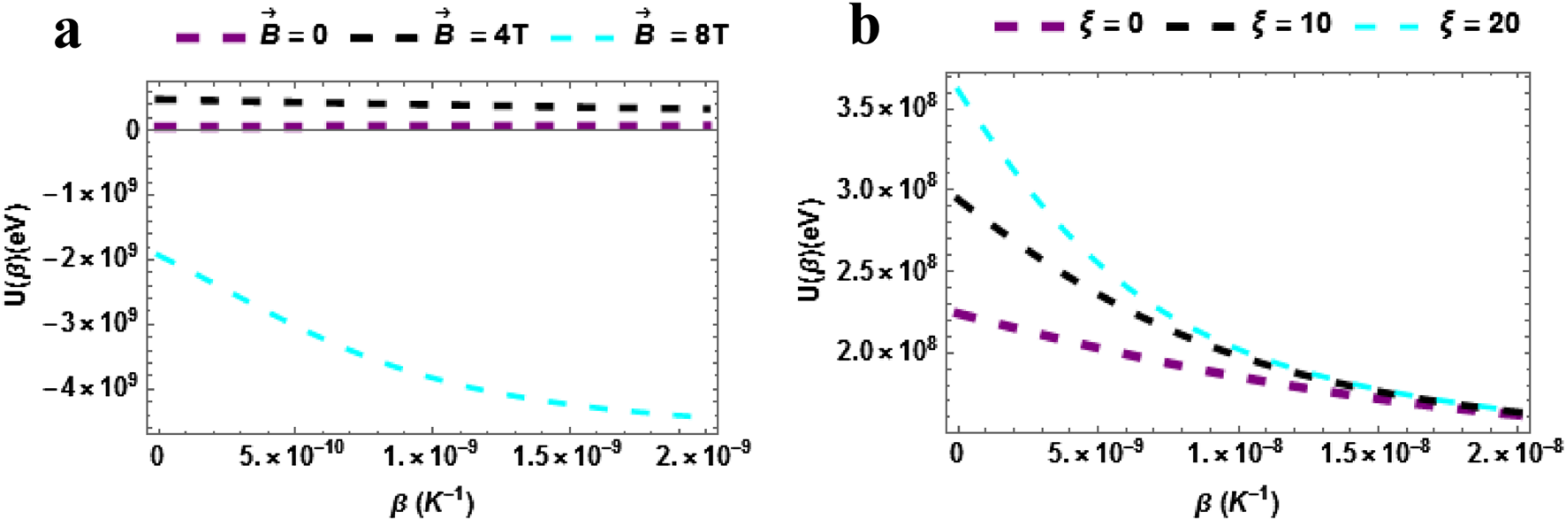
(**a**) Partition as a function of $\beta$ varying magnetic field (**b**) Partition as a function of $\beta$ varying AB field.

The internal energy also known as the average energy of the $TiH$ is plotted against a varying $\beta \left( {K^{ - 1} } \right)$ with different values of $\vec{B}$ in Fig. 4a. The internal energy decreases monotonically with rising temperature when $\vec{B} = 8T$. In the case where $\vec{B} = 0\,\& \,\vec{B} = 4T$, it is seen that the internal energy of the $TiH$ diatomic molecule is invariant but is higher than the case of $\vec{B} = 8T$. Again, a small amount of magnetic field is required to raise the internal energy. More so, it can be seen that when the magnetic field relatively low the influence is uniform as the influence shows an equally spaced internal energy profile when $\vec{B} = 0\,\& \,\vec{B} = 4T$. The average energy of the $TiH$ is plotted against a varying $\beta \left( {K^{ - 1} } \right)$ with different values of $\xi$ in Fig. 4b. The internal energy declines with rising temperature when $\xi = 0,\,\,\xi = 10\,T\,\& \xi = 20$. In addition, it can be seen that when the AB field rises, the internal energy rises and it shows a uniform pattern.

**Figure 5:**
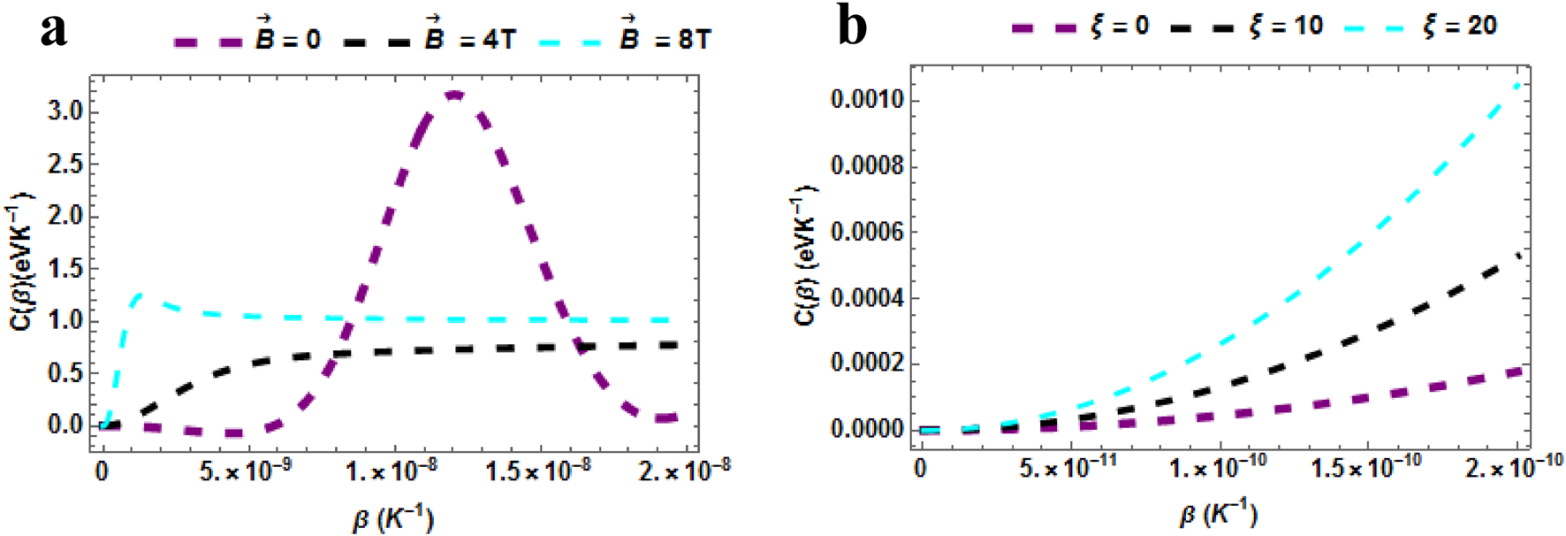
(**a**) Specific Heat Capacity as a function of $\beta$ varying magnetic field (**b**) Specific Heat Capacity as a function of $\beta$ varying AB field.

The specific heat capacity of the $TiH$ diatomic molecule plotted against $\beta \left( {K^{ - 1} } \right)$ with varying $\vec{B}$ in Fig. 5a. When $\vec{B} = 0\,$, it seen that the specific first rises and then reduces in a wave-like manner. Similar behaviour is observed when $\vec{B} = 4\,T\& \,\vec{B} = 8T$. Generally, it is seen that the specific heat capacity exhibits an irregular behaviour which is almost contrary to the fundamental Dulong-Petit law^9^. This anomaly could be attributed to the Schottky anomaly which appears over a small range of temperatures^65,66^. The observation of this Schottky anomaly indicates that there are small number of discrete energy levels dominating the behaviour of the TiH diatomic molecule, and the spacing between these energy levels can be quantified. The specific heat capacity of the $TiH$ diatomic molecule plotted against $\beta \left( {K^{ - 1} } \right)$ with varying $\xi$ in Fig. 5b. The specific heat upsurges with rising temperature with varying $\xi = 0,\,\,\xi = 10\,T\,\& \xi = 20$. It also rises with the rising AB field. In general, it is seen that the specific heat capacity exhibits a behaviour which is in consonance with Dulong-Petit law^9,43^.

**Figure 6:**
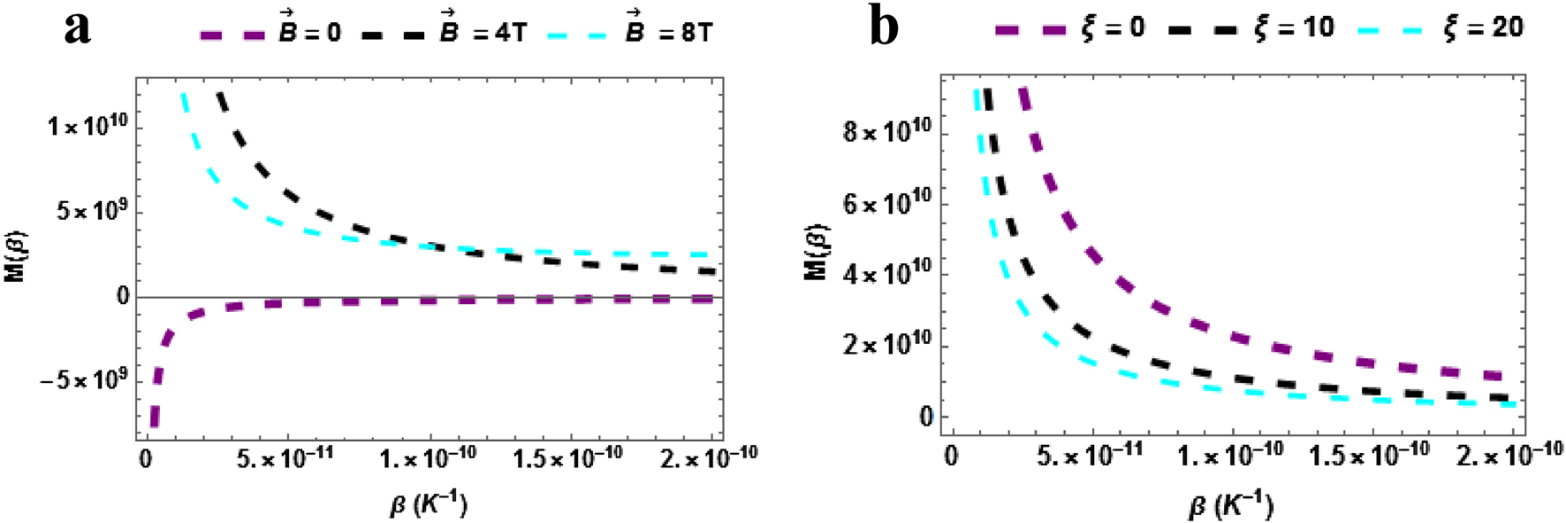
(**a**) Magnetization as a function of $\beta$ varying magnetic field (**b**) Magnetization as a function of $\beta$ varying AB field. Both for $\beta \ne 0$.

The magnetization of the $TiH$ diatomic molecule is plotted against temperature in Fig. 6a. The magnetization decreases with increasing temperature when $\vec{B} = 4\,T\& \,\vec{B} = 8T$. But increases with temperature monotonically when $\vec{B} = 0$. Moreover, the magnetization is higher when $\vec{B} = 4\,T\& \,\vec{B} = 8T$ even though the trend is a declining one. It can be inferred that the magnetization can be raised high when the magnetic field is intense. The reason for this decrease in magnetization with temperature increase is attributed to the fact that thermal disorder (kT) increases and opposes the magnetic dipoles of TiH to align with the applied magnetic field, this leads to a decreased magnetization. The magnetization of the $TiH$ diatomic molecule is plotted against temperature with varying AB field in Fig. 6b. The magnetization increases with increasing temperature when $\xi = 0,\,\,\xi = 10\,T\,\& \xi = 20$. But decreases with the rising AB field. It can be implied that the magnetization can be raised high when the AB field is low.

**Figure 7:**
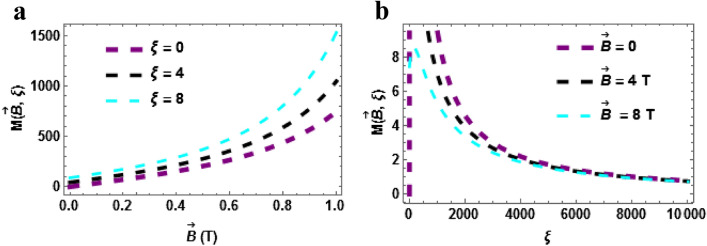
(**a**) Magnetization as a function of $\vec{B}\left( T \right)$ varying $\xi$-field (**b**) Magnetization as a function of $\xi$ varying $\vec{B}\left( T \right)$. Both for $\beta = 0$.

In Fig. 7a,b, the plot of the magnetization at zero temperature $\left( {\beta = 0} \right)$ are presented. In Fig. 7a, the magnetization is plotted against $\vec{B}\left( T \right)$ with a varying Aharonov-Bohm (AB) field $\left( \xi \right)$. The magnetization upsurges with the rising magnetic field and it also rise with different increasing values of the AB field. Figure 7b shows the magnetization plotted against $\xi$ with varying magnetic field. The magnetization declines with the rising AB field. The magnetization of the TiH molecules tends to be higher in the absence of the magnetic or at low magnetic field. This is similar to what was observed when $\beta \ne 0$.

**Figure 8:**
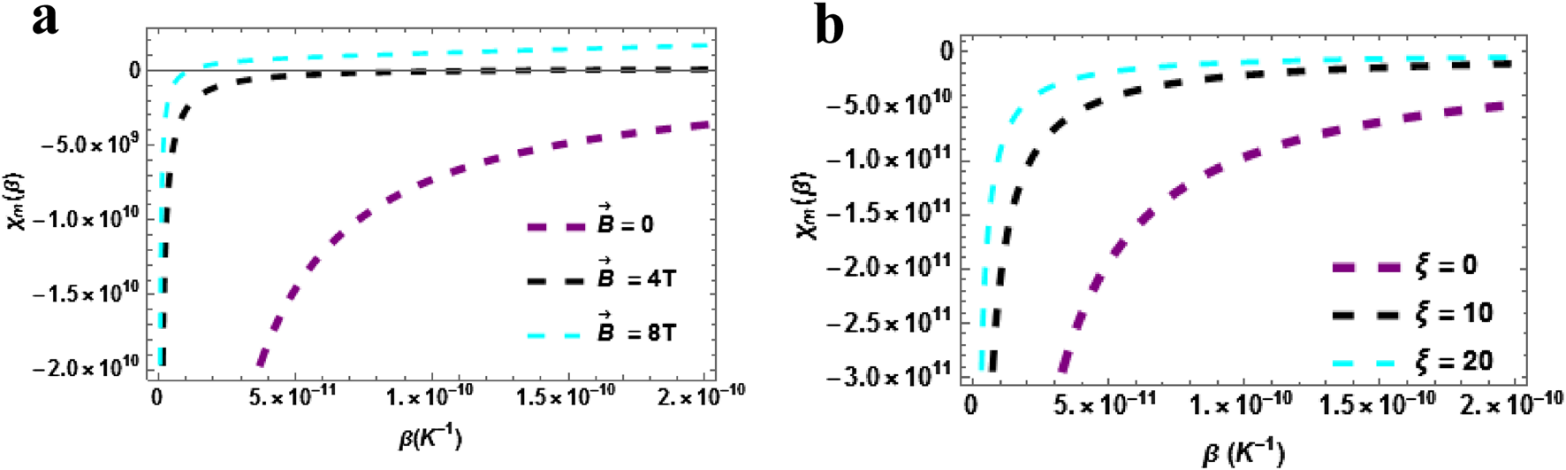
(**a**) Magnetic Susceptibility as a function of $\beta$ varying magnetic field (**b**) Magnetic Susceptibility as a function of $\beta$ varying AB field. Both for $\beta \ne 0$.

**Figure 9:**
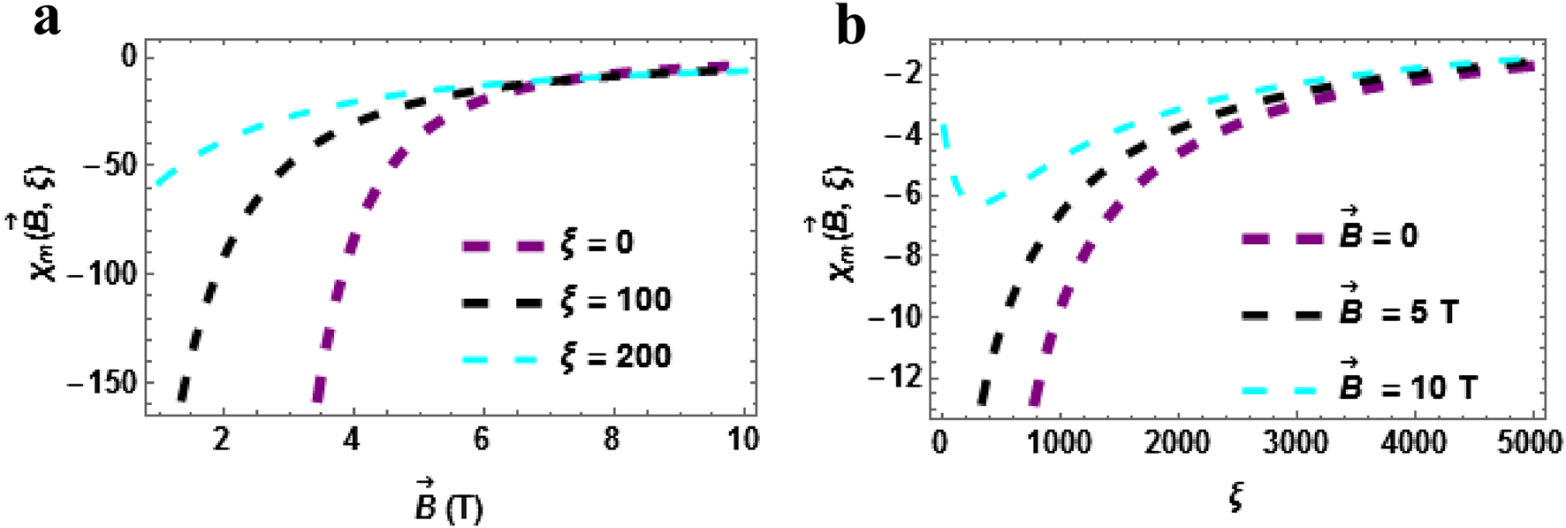
(**a**) Magnetic Susceptibility as a function of $\vec{B}\left( T \right)$ varying $\xi$-field (**b**) Magnetic Susceptibility as a function of $\xi$ varying $\vec{B}\left( T \right)$. Both for $\beta = 0$.

Magnetic susceptibility versus temperature with the varying magnetic field is plotted in Fig. 8a. The magnetic susceptibility upsurges with rising temperature in a monotonic pattern and this is uniform for all the cases considered $\vec{B} = 0,\,\,4\,T\& \,\vec{B} = 8T$. From the plot, it can be deduced that the magnetic susceptibility will rise higher when the magnetic field is intense. Magnetic susceptibility versus temperature with varying AB field $\left( {\xi = 0,\,\,\xi = 10\,T\,\& \xi = 20} \right)$ is plotted in Fig. 8b. The magnetic susceptibility upsurges with rising temperature in a monotonic pattern and this is uniform for all the cases considered $\xi = 0,\,\,\xi = 10\,T\,\& \xi = 20$. From the plot, it can be deduced that the magnetic susceptibility will rise higher when the AB field is intense. In Fig. 9a,b, the plot of the magnetic susceptibility at zero temperature $\left( {\beta = 0} \right)$ are presented. Magnetic susceptibility versus $\vec{B}\left( T \right)$ with varying AB field $\left( {\xi = 0,\,\,\xi = 100\,T\,\& \xi = 200} \right)$ is plotted in Fig. 9a. The magnetic susceptibility upsurges with rising temperature in a monotonic pattern and this is uniform for all the cases considered. More so, the susceptibility is higher when the AB field is absent or low. The magnetic susceptibility against AB field with varying magnetic field is plotted in Fig. 9b. The magnetic susceptibility increases with AB field and magnetic field. We point out here that in all the cases considered $\left( {\beta = 0\,\,\& \,\beta \ne 0} \right)$, the susceptibility of the TiH diatomic shows a diamagnetic behaviour since $\chi \left( {\vec{B},\xi } \right) < 0$.

**Figure 10:**
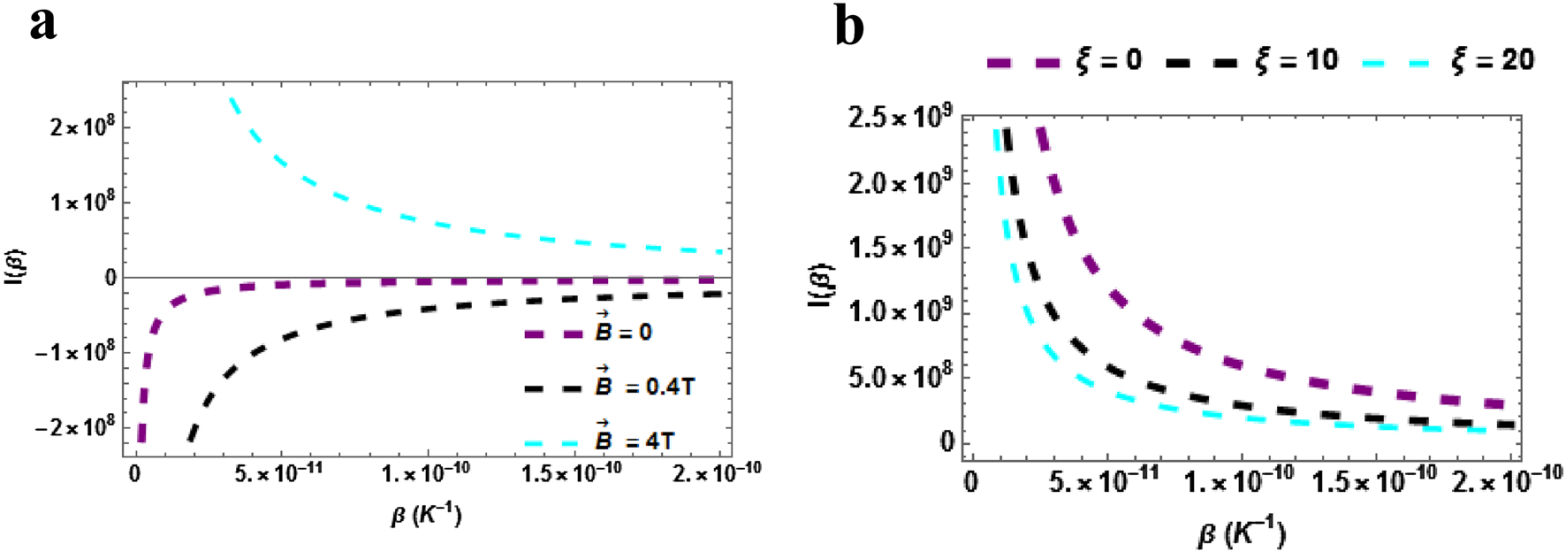
(**a**) Persistent current as a function of $\beta$ varying magnetic field (**b**) Persistent current as a function of $\beta$ varying AB field. Both for $\beta \ne 0$.

The persistent current (PC) of the $TiH$ diatomic molecule is plotted against temperature in Fig. 10a. The persistent current decreases with increasing temperature when $\vec{B} = 4T$. The PC increases with temperature monotonically when $\vec{B} = 0.4\,T\& \,\vec{B} = 4T$. Moreover, the persistent current is higher when $\vec{B} = 4\,T$ even though the trend is a declining one. It can be concluded that the persistent current can be raised high when the magnetic field is intense. The persistent current of the $TiH$ diatomic molecule is plotted against temperature with varying AB field $\left( {\xi = 0,\,\,\xi = 10\,T\,\& \xi = 20} \right)$ in Fig. 10b. The persistent current decreases monotonically with increasing temperature. In addition, the persistent current is higher when AB field is absent and low even though the trend is a declining one. It can be concluded that the persistent current can be raised high when the magnetic field is less-intense and absent.

**Figure 11:**
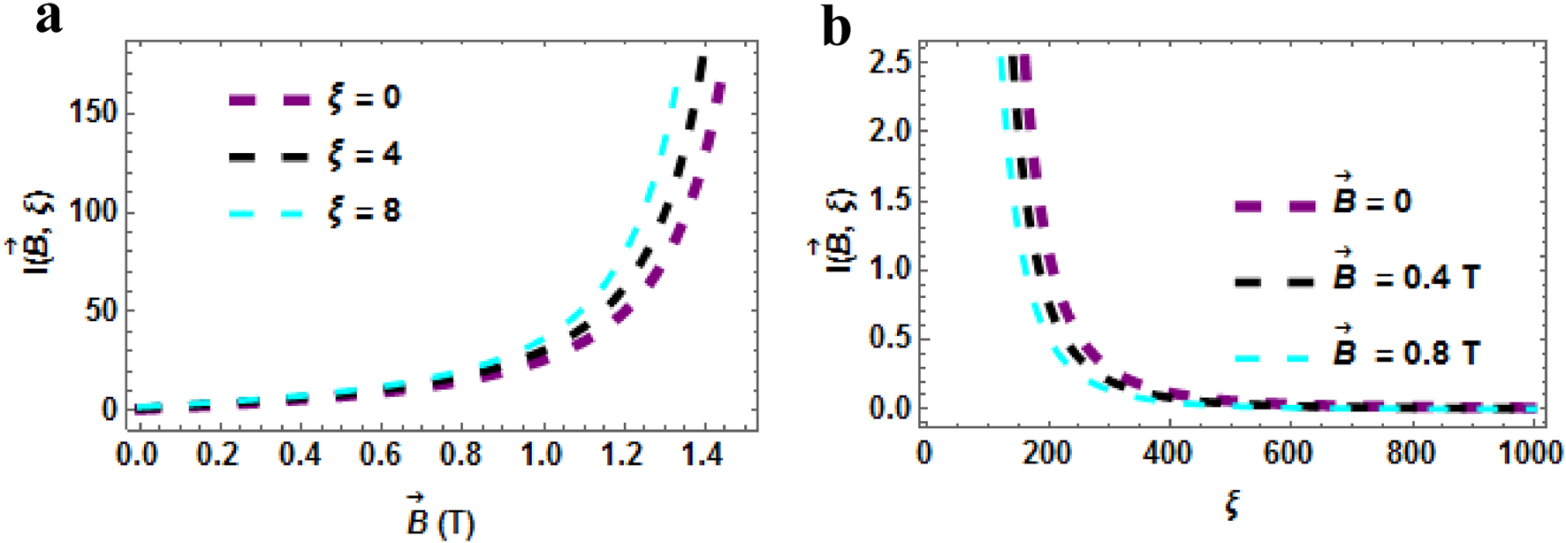
(**a**) Persistent current as a function of $\vec{B}\left( T \right)$ varying $\xi$-field (**b**) Persistent current as a function of $\xi$ varying $\vec{B}\left( T \right)$. Both for $\beta = 0$.

In Fig. 11a,b, the plot of the persistent current at zero temperature $\left( {\beta = 0} \right)$ are presented. Figure 11a shows the plot of the persistent current as a function of $\vec{B}$ with varying AB field. The persistent current rises with rising magnetic and AB fields (in a uniform trend). We point out here that our understanding of the persistent current in diatomic molecular systems is far from complete, especially at finite temperatures. We note here again that the current can change its flux period and sign (diamagnetic or paramagnetic) as a function of temperature, features that can be attributed to changing confinement of the system. This work presents the properties of the persistent current of TiH which could be relevant for the interpretation of experiments on persistent currents in such diatomic molecules. Figure 11b shows the plot of the persistent current as a function of $\xi$. The persistent current decreases with rising AB field. The persistent current is higher in the absence or region of low magnetic field.

**Figure 12:**
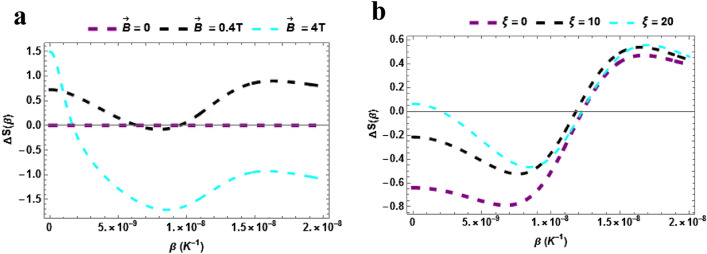
(**a**) Magnetic entropy as a function of $\beta$ varying magnetic field (**b**) Magnetic entropy as a function of $\beta$ varying AB field. Both for $\beta \ne 0$.

In Fig. 12a,b, the Magnetocaloric effect (MCE) of TiH is presented. The Magnetic entropy versus temperature with varying $\vec{B}$ is presented in Fig. 12a. The magnetic entropy shows a decrease with rising temperature when $\vec{B}\, = 4\,T$. There is no discernible trend at $\vec{B}\, = 0\,\,\& \,0.4\,T$. These findings could find possible applications in the fabrication of conventional cooling systems (air conditioners, refrigerators, and chillers) based on the MCE. These materials play an important role as coolants in adiabatic demagnetization refrigerators. The magnetic entropy versus temperature with varying $\xi$ is presented in Fig. 12b. The magnetic entropy rises with rising temperature and the influence of the various of values of the AB field is seen to be uniform.

## Conclusion

This work presents the thermal and magnetic properties of the TiH modelled by the Deng-Fan potential in the presence of magnetic and AB fields. The SE is solved using the FAA to obtain the approximate-analytic energy spectrum and wave function in terms of hypergeometric functions. The obtained energy equation is used to derive the analytic expressions for the thermo-magnetic properties. Graphical analysis are carried out extensively to show the effects of the perturbations on the thermo-magnetic and transport properties. We have shown in this work that the system is highly sensitive to the presence of the Aharonov-Bohm field and behaves in an irregular pattern when subjected to the magnetic field. Our study revealed that the susceptibility is diamagnetic which is in agreement with literature. By adjusting the potential parameters, the obtained analytical expressions can be used to study other physical systems, further expanding the frontiers of knowledge. Chemical physics, condensed matter physics, atomic physics, and other fields will benefit from the findings of this research. For instance, in chemical and condensed matter physics, several potential models (such as exponential-type potential considered in our study) have been adopted to study energy spectra and thermodynamics properties of diatomic molecules and GaAs quantum dot respectively by several researchers^59,67^ (and the references therein). In view of the foregoing, our results could be applied to study such systems highlighted above bearing in mind the effects external fields which was hitherto not considered.

## Data Availability

The datasets used and/or analyzed during the current study are available from the corresponding author on reasonable request.
